# *OAS1* Polymorphisms Are Associated with Susceptibility to West Nile Encephalitis in Horses

**DOI:** 10.1371/journal.pone.0010537

**Published:** 2010-05-07

**Authors:** Jonathan J. Rios, JoAnn G. W. Fleming, Uneeda K. Bryant, Craig N. Carter, John C. Huber, Maureen T. Long, Thomas E. Spencer, David L. Adelson

**Affiliations:** 1 McDermott Center for Human Growth and Development, University of Texas Southwestern Medical Center, Dallas, Texas, United States of America; 2 Department of Animal Science, Texas A&M University, College Station, Texas, United States of America; 3 Livestock Disease Diagnostic Center, University of Kentucky, Lexington, Kentucky, United States of America; 4 School of Rural Public Health, Texas A&M University, College Station, Texas, United States of America; 5 College of Veterinary Medicine, University of Florida, Gainesville, Florida, United States of America; 6 School of Molecular and Biomedical Science, The University of Adelaide, Adelaide, South Australia, Australia; Massachusetts General Hospital and Harvard Medical School, United States of America

## Abstract

West Nile virus, first identified within the United States in 1999, has since spread across the continental states and infected birds, humans and domestic animals, resulting in numerous deaths. Previous studies in mice identified the *Oas1b* gene, a member of the OAS/RNASEL innate immune system, as a determining factor for resistance to West Nile virus (WNV) infection. A recent case-control association study described mutations of human *OAS1* associated with clinical susceptibility to WNV infection. Similar studies in horses, a particularly susceptible species, have been lacking, in part, because of the difficulty in collecting populations sufficiently homogenous in their infection and disease states. The equine *OAS* gene cluster most closely resembles the human cluster, with single copies of *OAS1*, *OAS3* and *OAS2* in the same orientation. With naturally occurring susceptible and resistant sub-populations to lethal West Nile encephalitis, we undertook a case-control association study to investigate whether, similar to humans (*OAS1*) and mice (*Oas1b*), equine *OAS1* plays a role in resistance to severe WNV infection. We identified naturally occurring single nucleotide mutations in equine (*Equus caballus*) *OAS1* and *RNASEL* genes and, using Fisher's Exact test, we provide evidence that mutations in equine *OAS1* contribute to host susceptibility. Virtually all of the associated *OAS1* polymorphisms were located within the interferon-inducible promoter, suggesting that differences in *OAS1* gene expression may determine the host's ability to resist clinical manifestations associated with WNV infection.

## Introduction

The innate immune response confers host resistance by the recognition and limitation of viral infection and replication. Previous investigations of the innate immune response to West Nile virus (WNV) infection were conducted using inbred strains of naturally susceptible and resistant mice [1], [2]. Using a positional cloning strategy, the Flavivirus resistance (*Flv*) gene was identified as 2′,5′-oligoadenylate synthetase 1b (*Oas1b*). The interferon (IFN)-induced *OAS* genes encode dsRNA-activated proteins which catalyze the synthesis of 2′-5′-linked oligoadenylate molecules (2-5A) from ATP [3]–[5]. The only known function of 2-5A molecules is to activate Ribonuclease L (RNASEL) for the degradation of cellular and viral RNA [3], [6]–[8].

OAS proteins are encoded by multiple genes, collectively referred to as the *OAS* gene cluster. Gene clusters vary between species in both number of genes and splice variants. The rodent *Oas1* locus expanded to a family of 12 genes, while both the canine and bovine clusters contain duplications of *OASL* and *OAS1* genes, respectively [9], [10]. However, both equine and human *OAS* clusters contain single copies of each gene in the same orientation, *OAS1*-*OAS3*-*OAS2* and a single *OASL* gene [9], [11].

Previously, *Yakub et al.* found a SNP in human *OASL* associated with WNV susceptibility; however, this association was not replicated in a larger case-control study [12], [13]. Case-control association studies are best implemented using homogenous populations, which reduces systematic bias from sample selection and minimizes the potential for false positive associations inherent within the population structures [14]. *Lim et al.* recently identified human *OAS1* SNP rs10774671 in an association study of symptomatic and asymptomatic seroconverters [12]. In this study, case samples were compared to a control population of WNV false-positives. This SNP is located in an intron 5 splice site resulting in differential splicing and a protein product with diminished enzymatic activity. Taken together, data from both human and mouse studies support our investigation of equine candidate genes *OAS1* and *RNASEL*.

Susceptibility to severe West Nile encephalitis among mammals is naturally variable [15]. Experimental infections in sheep [16], calves [17], pigs [18], and dogs [19] have shown these domestic species to be poor hosts for, or develop only mild clinical symptoms from WNV infection, thereby limiting their usefulness for genetic susceptibility/resistance studies. Horses however, are particularly susceptible to severe WNV infection, suffering clinical symptoms including fever, ataxia, paralysis and death [20]. Because many horses infected with WNV remain asymptomatic or present only mild symptoms, horses are an excellent model organism to test for genetic susceptibility using strictly phenotyped case and control populations. This advantage is complicated, however, by the inaccessibility of well-characterized case and control samples for retrospective study.

As mentioned above, one advantage of a horse model is the ability to monitor both infection in control samples and WNV-induced encephalitis in case samples. In this report, we describe a two-stage association study of naturally occurring equine *OAS1* and *RNASEL* mutations to investigate a potential role of these genes in the equine innate immune response to WNV infection. Because of the limited retrospective accessibility to adequately phenotyped samples with known pre- and post-infection status, our population sizes did not allow matching of case and control samples by breed. Although no breed-specific difference in susceptibility has been reported, our analysis showed the associations identified from these populations were not artifacts of the two most frequent breeds in our case population. Because most significantly associated SNPs were present in the *OAS1* promoter region, we conducted reporter assays to measure the response of equine *OAS1* promoter constructs to interferon stimulation by transient transfection using human transformed cell lines.

## Results

### Defining case and control population samples

The control population consisted of 16 healthy, previously uninfected (naïve) horses of multiple breeds, including Thoroughbred (13), Quarterhorse (1), Paso Fino (1) and a single mixed breed horse. These unvaccinated horses were naturally infected with WNV by mosquito transmission during the height of the initial Florida epidemic of 2001, when only the NY99 strain was present. Control horses were monitored daily, yet failed to exhibit clinical symptoms. These healthy individuals tested positive for WNV infection and were therefore classified as subclinical seroconverters. An important characteristic of our case-control study, all horses included in the control population had an equal opportunity of being classified in the case population, had they displayed clinical symptoms post-infection.

Horses included in the case population were previously unvaccinated and naturally infected through mosquito transmission. Multiple breeds were present among the 44 case horses, the most common of which included Thoroughbred (12) and Quarterhorse (10), with 2 horses of unknown breed. All case horses developed clinical encephalitic symptoms diagnosed with veterinary treatment, ultimately requiring humane euthanasia. Veterinary examination noted multiple symptoms, the most common including forelimb and/or hindlimb ataxia. Diagnostic tests confirmed WNV infection by enzyme-linked immunosorbent assay (ELISA) or polymerase chain reaction (PCR).

An important characteristic shared by both the case and control populations, horses were unvaccinated prior to infection and all horses were infected by natural mosquito transmission during the early stages of the initial U.S. epidemic, between 2001 and 2002.

### SNP genotyping and association analysis

Previously, we sequenced the coding regions of equine *OAS1* and *RNASEL* as well as the *OAS1* promoter from a random population and identified SNPs for case-control association [21]. In this study, we genotyped 49 equine *OAS1* and *RNASEL* mutations in 16 control and 44 case samples. Genotype data were analyzed to identify statistically significant allelic (2×2) and genotypic (2×3) associations to WNV phenotype using the conservative Fisher's Exact test. Fifteen SNPs in *OAS1* and three SNPs in exon 2 of *RNASEL* were significantly associated (2×2, p<0.05) with WNV susceptibility (Table 1). The statistical associations of *OAS1* mutations were not uniformly scattered throughout the entire gene but were concentrated in the upstream regulatory region, with twelve of the significantly associated mutations located in the *OAS1* promoter and 5′ untranslated region (UTR). Using the highly conservative Bonferonni threshold for statistical significance, only mutations of the *OAS1* promoter (6 of 49) were associated (2×2, p<0.001) with WNV susceptibility. Next, we used odds ratios (OR) to measure the strength of the SNP associations (Table 1). All significantly associated mutations had ORs greater than 1.0. Among significantly associated mutations of the *OAS1* regulatory region, all but one had 95% OR confidence intervals greater than 1.0.

**Table 1:** Statistical association of single nucleotide polymorphisms to West Nile encephalitis.

SNP ID	Gene	Region	Allele	Case Allele Frequency	Control Allele Frequency	Fisher Exact 2×3	Fisher Exact 2×2	Odds Ratio	Odds Ratio 95% CI
ss104806917	OAS1	Promoter	C	0.60	0.20	0.0001	0.0002	5.844	2.043; 19.437
ss104806918	OAS1	Promoter	T	0.57	0.20	3.118e-5	0.0006	5.256	1.846; 17.405
ss104806919	OAS1	Promoter	G	0.40	0.19	0.3181	0.0305	2.921	1.030; 9.621
ss104806920	OAS1	Promoter	T	0.54	0.19	9.465e-6	0.0008	4.933	1.750; 16.210
ss104806921	OAS1	Promoter	C	0.51	0.19	0.0001	0.0016	4.488	1.592; 14.720
ss104806922	OAS1	Promoter	T	0.33	0.13	0.0072	0.0351	3.468	1.060; 14.934
ss104806923	OAS1	Promoter	T	0.53	0.19	2.676e-5	0.0008	4.918	1.750; 16.123
ss104806924	OAS1	Promoter	T	0.55	0.19	0.0003	0.0007	5.151	1.832; 16.900
ss104806925	OAS1	Promoter	G	0.98	0.88	0.0494	0.0546	5.470	0.738; 63.646
ss104806926	OAS1	Promoter	T	0.66	0.19	1.227e-5	6.95e-6	8.189	2.869; 27.277
ss104806927	OAS1	Promoter	G	0.97	0.84	0.0563	0.0232	6.709	0.023; 0.012
ss104806928	OAS1	5′UTR	G	0.54	0.17	0.0356	0.0017	5.773	1.772; 22.631
ss104806929	OAS1	5′UTR	A	0.50	0.19	0.0705	0.0082	4.252	1.372; 15.087
ss104806930	OAS1	Exon 1	G	0.10	0.06	1.0000	0.6962	1.733	0.262; 19.333
ss104806931	OAS1	Exon 1	C	0.44	0.19	0.1304	0.0196	3.418	1.132; 11.840
ss104806932	OAS1	Intron 1	T	0.22	0.28	0.7197	0.4745	0.721	0.261; 2.090
ss104806933	OAS1	Exon 2	T	0.31	0.28	0.4466	0.8213	1.151	0.428; 3.285
ss104806934	OAS1	Exon 2	T	0.84	0.72	0.1275	0.1894	2.003	0.663; 5.874
ss104806935	OAS1	Exon 2	A	0.83	0.72	0.1487	0.1949	1.944	0.642; 5.707
ss104806936	OAS1	Exon 2	G	0.84	0.72	0.1275	0.1894	2.003	0.663; 5.874
ss104806937	OAS1	Exon 2	G	0.83	0.72	0.1734	0.2996	1.834	0.613; 5.295
ss104806938	OAS1	Exon 4	C	0.73	0.47	0.0456	0.0144	3.042	1.196; 7.893
ss104806939	OAS1	Intron 4	C	0.63	0.47	0.2948	0.1746	1.952	0.733; 5.296
ss104806940	OAS1	Intron 4	G	0.73	0.43	0.0849	0.0098	3.490	1.244; 10.189
ss104806941	OAS1	Intron 4	G	0.51	0.33	0.4243	0.1262	2.102	0.802; 5.803
ss104806942	OAS1	Exon 5	G	0.41	0.17	0.2279	0.0538	3.432	0.960; 14.328
ss104806943	OAS1	Intron 5	T	0.54	0.43	0.3564	0.3824	1.554	0.603; 4.082
ss104806944	OAS1	Intron 5	G	0.51	0.43	0.6787	0.5150	1.382	0.537; 3.626
ss104806945	OAS1	Exon 6	T	0.38	0.27	0.3278	0.3480	1.641	0.572; 5.062
ss104806946	OAS1	3′UTR	T	0.38	0.53	0.1535	0.2407	0.529	0.188; 1.462
ss104806947	RNASEL	Exon 2	C	0.57	0.31	0.1809	0.0351	2.889	1.013; 8.694
ss104806948	RNASEL	Exon 2	A	0.88	0.69	0.0688	0.0464	3.263	0.977; 11.549
ss104806949	RNASEL	Exon 2	T	0.35	0.38	0.9186	0.8232	0.898	0.338; 2.434
ss104806950	RNASEL	Exon 2	C	0.85	0.69	0.1197	0.1030	2.547	0.806; 8.212
ss104806951	RNASEL	Exon 2	G	0.30	0.28	1.0000	1.0000	1.094	0.389; 3.234
ss104806952	RNASEL	Exon 2	C	0.26	0.38	0.5506	0.2470	0.583	0.212; 1.616
ss104806953	RNASEL	Exon 2	C	0.78	0.72	0.1766	0.6119	1.350	0.439; 4.023
ss104806954	RNASEL	Exon 2	C	0.26	0.22	0.8220	0.8068	1.277	0.440; 4.079
ss104806955	RNASEL	Exon 2	G	0.61	0.44	0.2420	0.1345	2.006	0.800; 5.135
ss104806956	RNASEL	Exon 2	G	0.81	0.86	0.2621	0.7673	0.697	0.148; 2.625
ss104806957	RNASEL	Exon 2	G	0.67	0.82	0.3144	0.1448	0.438	0.115; 1.401
ss104806958	RNASEL	Exon 2	G	0.38	0.25	0.4311	0.3308	1.821	0.615; 5.933
ss104806959	RNASEL	Exon 2	A	0.55	0.21	0.0267	0.0035	4.465	1.533; 15.032
ss104806960	RNASEL	3′UTR	G	0.87	0.86	0.8300	1.0000	1.110	0.208; 5.240
ss104806961	RNASEL	3′UTR	A	0.89	0.79	0.4864	0.3070	2.030	0.413; 10.025
ss104806962	RNASEL	3′UTR	T	0.83	0.75	0.7527	0.3765	1.657	0.445; 5.643
ss104806963	RNASEL	3′UTR	C	0.41	0.25	0.2983	0.2190	2.061	0.670; 7.195
ss104806964	RNASEL	3′UTR	T	0.17	0.12	1.0000	0.7410	1.596	0.351; 10.058
ss104806965	RNASEL	3′UTR	T	0.67	0.73	0.7255	0.6159	0.740	0.221; 2.281

Deviation from Hardy-Weinberg Equilibrium (HWE) can be a useful tool to indicate errors in genotyping or population stratification [22]. To investigate this possibility, exact tests were used to determine if genotype frequencies in the control population deviated from HWE. Two SNPs (ss104806941 and ss104806963) failed the HWE test (Table 2), but neither SNP was associated with WNV susceptibility. Tests of HWE may also be informative for association studies when measured in the case population. Within our case population, many SNPs deviated significantly (p<0.05) from HWE (Table 2), and SNPs with the greatest deviation (lowest p-value) were in the *OAS1* promoter. Exact tests for HWE in the control and case populations supported the significant associations of 6 of the 10 *OAS1* promoter mutations.

**Table 2:** Exact test of HWE among equine *OAS1* and *RNASEL* SNPs.

SNP ID	Gene	Region	Control HWE p-value	Case HWE p-value
ss104806917	OAS1	Promoter	0.4605	0.1179
ss104806918	OAS1	Promoter	0.4605	0.0048
ss104806919	OAS1	Promoter	0.4344	0.0003
ss104806920	OAS1	Promoter	0.4344	0.0004
ss104806921	OAS1	Promoter	0.4344	0.0047
ss104806922	OAS1	Promoter	1.000	0.0012
ss104806923	OAS1	Promoter	0.4344	0.0021
ss104806924	OAS1	Promoter	0.4344	0.1265
ss104806925	OAS1	Promoter	1.000	3.2785 e-9
ss104806926	OAS1	Promoter	0.4344	0.3088
ss104806927	OAS1	Promoter	0.3059	1.0000
ss104806928	OAS1	5′UTR	0.3257	0.0165
ss104806929	OAS1	5′UTR	0.4344	0.0914
ss104806930	OAS1	Exon 1	1.0000	0.2057
ss104806931	OAS1	Exon 1	0.4344	0.0500
ss104806932	OAS1	Intron 1	1.0000	0.3651
ss104806933	OAS1	Exon 2	1.0000	0.0652
ss104806934	OAS1	Exon 2	1.0000	0.0449
ss104806935	OAS1	Exon 2	1.0000	0.0483
ss104806936	OAS1	Exon 2	1.0000	0.0449
ss104806937	OAS1	Exon 2	1.0000	0.0727
ss104806938	OAS1	Exon 4	0.6376	0.0986
ss104806939	OAS1	Intron 4	0.6376	0.0378
ss104806940	OAS1	Intron 4	0.2939	0.0440
ss104806941	OAS1	Intron 4	0.0101	3.1126 e-6
ss104806942	OAS1	Exon 5	0.3257	0.0452
ss104806943	OAS1	Intron 5	0.2939	1.0000
ss104806944	OAS1	Intron 5	0.2939	0.5044
ss104806945	OAS1	Exon 6	1.0000	0.0161
ss104806946	OAS1	3′UTR	0.6035	0.3916
ss104806947	RNASEL	Exon 2	0.1068	0.3771
ss104806948	RNASEL	Exon 2	0.5914	1.0000
ss104806949	RNASEL	Exon 2	0.5914	0.1016
ss104806950	RNASEL	Exon 2	0.5914	1.0000
ss104806951	RNASEL	Exon 2	0.5301	0.0730
ss104806952	RNASEL	Exon 2	1.0000	0.3682
ss104806953	RNASEL	Exon 2	1.0000	0.0134
ss104806954	RNASEL	Exon 2	0.1081	0.0003
ss104806955	RNASEL	Exon 2	0.3477	0.0015
ss104806956	RNASEL	Exon 2	1.0000	0.0042
ss104806957	RNASEL	Exon 2	1.0000	0.1101
ss104806958	RNASEL	Exon 2	1.0000	0.0435
ss104806959	RNASEL	Exon 2	0.0647	0.0261
ss104806960	RNASEL	3′UTR	0.2178	0.0229
ss104806961	RNASEL	3′UTR	0.4037	1.0000
ss104806962	RNASEL	3′UTR	0.0899	0.0352
ss104806963	RNASEL	3′UTR	0.0016	0.0001
ss104806964	RNASEL	3′UTR	1.0000	1.0000
ss104806965	RNASEL	3′UTR	0.1652	0.0007

To investigate potential false-positive SNP associations from over-represented case breeds, Quarterhorse (n = 10) and Thoroughbred (n = 12) case samples, together representing 50% of the population, were independently compared to the control population. Seven of the 8 mutations statistically significant in both case-breed analyses were in the equine *OAS1* promoter (Table 3). Using the two most represented breeds of the case population, these analyses allow us to conclude that the SNP associations to WNV susceptibility are not artifacts attributable to breed specific allele frequencies in the major breeds of our case study population.

**Table 3:** Quarterhorse and Thoroughbred breed case-control allelic Fisher's Exact analysis.

SNP ID	Gene	Region	Allele	Case Allele Freq.	Control Allele Freq.	Case Quarterhorse Allele Freq.	Case Quarterhorse Fisher Exact 2×2	Case Thoroughbred Allele Freq.	Case Thoroughbred Fisher Exact 2×2
ss104806917	OAS1	Promoter	C	0.60	0.20	0.65	0.0025	0.64	0.0033
ss104806918	OAS1	Promoter	T	0.57	0.20	0.60	0.0065	0.63	0.0021
ss104806919	OAS1	Promoter	G	0.40	0.19	0.45	0.0607	0.29	0.5237
ss104806920	OAS1	Promoter	T	0.54	0.19	0.50	0.0297	0.63	0.0018
ss104806921	OAS1	Promoter	C	0.51	0.19	0.55	0.0137	0.50	0.0205
ss104806922	OAS1	Promoter	T	0.33	0.13	0.30	0.1562	0.42	0.0268
ss104806923	OAS1	Promoter	T	0.53	0.19	0.50	0.0297	0.54	0.0096
ss104806924	OAS1	Promoter	T	0.55	0.19	0.50	0.0297	0.54	0.0096
ss104806925	OAS1	Promoter	G	0.98	0.88	1.00	0.1507	1.00	0.1368
ss104806926	OAS1	Promoter	T	0.66	0.19	0.65	0.0012	0.73	0.0002
ss104806927	OAS1	Promoter	G	0.97	0.84	1.00	0.1542	1.00	0.0720
ss104806928	OAS1	5′UTR	G	0.54	0.17	0.50	0.0852	0.50	0.0852
ss104806929	OAS1	5′UTR	A	0.50	0.19	0.50	0.0944	0.63	0.3476
ss104806930	OAS1	Exon 1	G	0.10	0.06	0.10	1.0000	0.00	1.0000
ss104806931	OAS1	Exon 1	C	0.44	0.19	0.75	0.7118	0.56	0.0900
ss104806932	OAS1	Intron 1	T	0.22	0.28	0.17	0.4973	0.21	0.7561
ss104806933	OAS1	Exon 2	T	0.31	0.28	0.39	0.5322	0.27	1.0000
ss104806934	OAS1	Exon 2	T	0.84	0.72	0.72	1.0000	0.92	0.0926
ss104806935	OAS1	Exon 2	A	0.83	0.72	0.33	0.7544	0.05	0.0358
ss104806936	OAS1	Exon 2	G	0.84	0.72	0.67	0.7544	0.96	0.0329
ss104806937	OAS1	Exon 2	G	0.83	0.72	0.67	0.7544	0.96	0.0329
ss104806938	OAS1	Exon 4	C	0.73	0.47	0.15	0.0083	0.17	0.0062
ss104806939	OAS1	Intron 4	C	0.63	0.47	0.42	0.7360	0.33	0.3182
ss104806940	OAS1	Intron 4	G	0.73	0.43	0.57	0.5206	0.83	0.0372
ss104806941	OAS1	Intron 4	G	0.51	0.33	0.63	0.0701	0.35	1.0000
ss104806942	OAS1	Exon 5	G	0.41	0.17	0.25	0.6236	0.17	1.0000
ss104806943	OAS1	Intron 5	T	0.54	0.43	0.56	0.5524	0.63	0.3534
ss104806944	OAS1	Intron 5	G	0.51	0.43	0.50	0.7676	0.63	0.3534
ss104806945	OAS1	Exon 6	T	0.38	0.27	0.42	0.4635	0.28	1.0000
ss104806946	OAS1	3′UTR	T	0.38	0.53	0.29	0.1948	0.36	0.3419
ss104806947	RNASEL	Exon 2	C	0.57	0.31	0.50	0.4162	0.58	0.1641
ss104806948	RNASEL	Exon 2	A	0.88	0.69	0.21	0.7241	0.00	0.0085
ss104806949	RNASEL	Exon 2	T	0.35	0.38	0.64	0.1088	0.33	1.0000
ss104806950	RNASEL	Exon 2	C	0.85	0.69	0.25	0.7460	0.00	0.0196
ss104806951	RNASEL	Exon 2	G	0.30	0.28	0.63	0.0306	0.25	1.0000
ss104806952	RNASEL	Exon 2	C	0.26	0.38	0.38	1.0000	0.17	0.1990
ss104806953	RNASEL	Exon 2	C	0.78	0.72	1.00	0.0406	0.71	1.0000
ss104806954	RNASEL	Exon 2	C	0.26	0.22	0.50	0.0963	0.27	0.7500
ss104806955	RNASEL	Exon 2	G	0.61	0.44	0.31	0.5352	0.63	0.1761
ss104806956	RNASEL	Exon 2	G	0.81	0.86	0.94	0.6342	0.67	0.1569
ss104806957	RNASEL	Exon 2	G	0.67	0.82	0.94	0.3920	0.61	0.1703
ss104806958	RNASEL	Exon 2	G	0.38	0.25	0.21	1.0000	0.36	0.4913
ss104806959	RNASEL	Exon 2	A	0.55	0.21	0.40	0.2061	0.45	0.1170
ss104806960	RNASEL	3′UTR	G	0.87	0.86	1.00	0.2969	0.58	0.0968
ss104806961	RNASEL	3′UTR	A	0.89	0.79	0.17	1.0000	0.10	0.6445
ss104806962	RNASEL	3′UTR	T	0.83	0.75	0.70	0.7456	0.90	0.2591
ss104806963	RNASEL	3′UTR	C	0.41	0.25	0.89	0.4307	0.44	0.0590
ss104806964	RNASEL	3′UTR	T	0.17	0.12	0.08	1.0000	0.33	0.1284
ss104806965	RNASEL	3′UTR	T	0.67	0.73	0.92	0.3934	0.50	0.2018

### Haplotype assembly and association analysis

Fifteen SNPs genotyped in the promoter, 5′UTR and exon 1 of equine *OAS1* were used to infer haplotypes among case and control samples. From the assembled best reconstruction, we identified six tagSNPs (ss104806918, ss104806922, ss104806924, ss104806926, ss104806927 and ss104806931) with calculated mean percentage diversity explained (PDE) of 99.23% [23]. These tagSNPs, all associated with WNV susceptibility, were used to re-construct haplotypes and conduct case-control comparisons. Haplotype frequencies were significantly different (p<0.01) between case and control populations. A single common haplotype (GACCGT) was assembled in 65.6% and 23.3% of control and case sample chromosomes, respectively. Fisher's Exact test showed deviations from this haplotype were significantly associated (p = 4.953 e-6) with susceptibility to severe WNV disease, with an odds ratio of 7.58 (95% CI = 2.88: 21.18). Five of the six alleles in this haplotype were found to be protective in our study, consistent with the increased haplotype frequency in the control population. This haplotype data supports the *OAS1* promoter SNP associations to WNV susceptibility.

Equine *RNASEL* haplotypes were inferred from 42 horses genotyped at ≥75% of all *RNASEL* SNPs in order to minimize the effect of unknown genotypes. Six tagSNPs (ss104806949, ss104806954, ss104806955, ss104806958, ss104806959 and ss104806965) were identified with total mean PDE of 99.33%. Haplotypes were re-constructed using these tagSNPs from the same 42 samples and, in contrast to *OAS1*, haplotype frequencies were not found to differ significantly between case and control populations (p = 0.53).

### Interferon stimulation of equine *OAS1* promoter

Since many of the SNPs associated with WNV susceptibility were present in the *OAS1* promoter and because human *OAS1* is induced by IFN-stimulated regulatory factors acting through an IFN-stimulated response element (ISRE) proximal to the transcription start site (TSS, Figure S1) [24], we conducted preliminary transient transfection experiments to determine if these mutations could alter IFN induction of the equine *OAS1* promoter.

Functional assays of the *OAS1* promoter by transient transfection should be conducted in equine cell lines derived from tissues involved in the early development of post-infection WNV disease, but such cell lines are currently unavailable. We therefore substituted two cell lines, 2fTGH and HepG2, that have been extensively used in studies of IFN and/or *OAS* [25]–[29].

**Figure 1:**
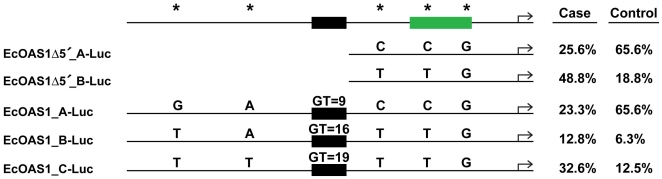
*OAS1*-Luciferase expression constructs, genotypes and population frequency. Schematic diagram of the *OAS1* promoter constructs expressing the luciferase reporter coding region. The interferon stimulated response element (green) is shown from sequence alignments between horse and human *OAS1* promoters. The previously identified dinucleotide microsatellite (black) is shown with corresponding repeat length. Deletion constructs EcOAS1Δ5′_A-Luc and EcOAS1Δ5′_B-Luc do not contain the microsatellite repeat and upstream sequence. TagSNPs(*) ss104806918, ss104806922, ss104806924, ss104806926, ss104806927 and genotypes are shown for each construct. Case and control haplotype frequencies represented by each clone are also shown.

Haplotypes of the proximal promoter of equine *OAS1* were cloned upstream of the luciferase reporter coding region (Figure 1). Proximal promoter constructs were generated as deletions of the full-length clones mentioned below. These deletion constructs (EcOAS1Δ5′_A-Luc and EcOAS1Δ5′_B-Luc) lack the polymorphic microsatellite and sequence further upstream. These proximal promoter constructs were used in transient transfection assays of 2fTGH cells (a derivative of HT1080 cells) treated with 10^4^ antiviral units (AVU) of interferon (IFN). Luciferase reporter activity 24 h after stimulation was 7- to 8-fold higher than basal levels (data not shown). Therefore, the proximal region from the TSS to the microsatellite (∼518 bp) was found to be necessary and sufficient for equine *OAS1* promoter responsiveness to IFN. This is the first direct observation of equine *OAS1* promoter IFN responsiveness.

Additional promoter sequence containing the polymorphic microsatellite and upstream sequence was cloned upstream of the luciferase reporter coding region (Figure 1). Full-length promoter clone EcOAS1_A-Luc contains the alleles of the common haplotype previously mentioned (GACCG) while EcOAS1_B-Luc and EcOAS1_C-Luc contain the TATTG and TTTTG haplotypes, respectively. Additionally, each full-length clone contains a previously identified polymorphic microsatellite with repeat lengths of 9 (A), 16 (B) and 19 (C) [21].

**Figure 2:**
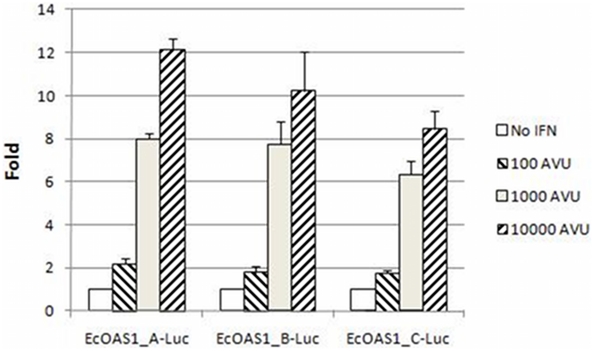
Effect of IFN dose on *OAS1*-luciferase activity in 2fTGH fibroblast cells. Cells were transfected with full-length clones and treated with 10^2^, 10^3^ or 10^4^ AVU IFN. Reporter activity was measured 24 hours after treatment in triplicate. All constructs showed a dose-response to IFN. Statistically significant differences in IFN response occurred when cells were treated with 10,000 AVU IFN (ANOVA, p = 0.026). Pair-wise comparison of EcOAS1_A-Luc and EcOAS1_C-Luc fold induction was statistically significant after Bonferroni correction (p = 0.0275).

Full-length constructs were transfected into 2fTGH cells and treated with different doses (10^2^ to 10^4^ AVU/mL) of IFN for 24 h. IFN stimulated activity of each *OAS* construct in a dose-dependent manner (Figure 2). The variation between experiments was greater for the EcOAS1_B-Luc construct; however, the average fold induction across three replicates for the EcOAS1_A-Luc construct was ∼2- and ∼4-fold greater than the EcOAS1_C-Luc construct when cells were treated with 10^3^ AVU and 10^4^ AVU, respectively. The greatest differences in fold induction between clones occurred when cells were treated with 10,000 AVU IFN (ANOVA, p = 0.026); however, little difference was seen between constructs when cells were treated with only 100 AVU for 24 h.

**Figure 3:**
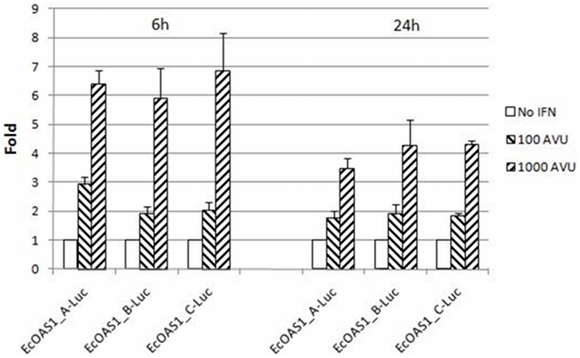
Effect of IFN on *OAS1*-luciferase activity in HepG2 cells. HepG2 cells were transfected with the full-length *OAS1* constructs and treated with 100 or 10^3^ AVU IFN for 6 h or 24 h. A dose-response was observed for all clones, averaged across 4 replicates. Treating cells with 10^3^ AVU IFN did not result in statistically significant differences in fold induction at either timepoint. However, when cells were treated with 100 AVU IFN, EcOAS1_A-Luc responded with greater fold induction than the other clones (ANOVA, p<0.001). Pair-wise comparisons of EcOAS1_A-Luc to EcOAS1_B-Luc (p = 0.001) and EcOAS1_C-Luc (p = 0.003) resulted in statistically significant differences in fold induction after Bonferroni multiple test correction.

Because the difference in fold induction between constructs may be an artifact of the high IFN concentration used and the long exposure time, full-length constructs were transfected into HepG2 liver cells, which have been used in previous studies of *OAS* expression, and treated for 6 h or 24 h [29]. Similar to the 2fTGH experiment, equine *OAS1* promoter induction in HepG2 cells showed a dose-response to IFN at both 6 h and 24 h (Figure 3). Averaged across 4 replicates, the fold induction was similar between clones both at 6 h and 24 h when cells were treated with 10^3^ AVU IFN (ANOVA, p>0.05). However, when HepG2 cells were treated with 100 AVU IFN for 6 h, construct EcOAS1_A-Luc was induced at higher fold levels than the other clones (ANOVA, p<0.001). *OAS* is part of the immediate early response of cells to viral infection, and previous studies of *OAS* promoter function have included lower IFN doses and shorter exposure periods to mimic early times after infection [28]. These preliminary data suggest a similar model, and when considered with the promoter SNP and haplotype associations, suggest promoter mutations affecting equine OAS1 expression may contribute, in part, to susceptibility to severe WNV disease.

## Discussion

The first evidence for an involvement of the *OAS* gene family in innate resistance to West Nile virus was provided using a mouse model [1], [2]. However, the rodent *OAS* cluster contains numerous copies of the *Oas1* gene and the comprehensive cluster structure is largely different from the human gene cluster. Another study recently provided evidence of a role for human OAS1 in innate resistance to WNV infection [12]. With our ability to closely monitor response to West Nile virus infection, we used the horse as a model to identify a potential association of the equine *OAS1* gene to WNV resistance or susceptibility through genetic comparisons of case (susceptible) and control (resistant) animals subjected to similar pre-exposure conditions. These horses were phenotyped for their innate resistance and susceptibility to natural WNV infection. Furthermore, the equine *OAS* gene cluster is more similar to the human *OAS* cluster than any other known cluster in domesticated mammals.

We report associations of SNPs in the equine *OAS1* gene with susceptibility to West Nile encephalitis. Because of the limited number of well-characterized horses, Fisher's Exact tests were used to identify significant differences in allelic and genotypic frequencies between case and control populations. Ten of the 18 susceptibility-associated SNPs (p<0.05) were identified within the regulatory region of equine *OAS1*. While these data suggest a potential role for equine *OAS1* in innate resistance to WNV disease, identifying causal mutations is complicated by the highly variable nature of this region within our case population and the possibility of as yet unidentified mutations in strong linkage disequilibrium with those reported here.

Although no difference in susceptibility to WNV infection between major equine breeds in the United States has been reported, we investigated the potential for false-positive associations resulting from the two most frequent breeds of our case population, Quarterhorse and Thoroughbred. Both breeds, together representing 50% of the case population, were individually compared to the control population which is almost entirely composed of Thoroughbreds. Promoter polymorphism associations remained significant for each breed, indicating that the associations reported here are not artifacts based on skewing from the major case population breeds.

While the multiple SNP associations of the *OAS1* promoter with WNV susceptibility are likely due to linkage disequilibrium, our association data also suggest a potential functional mechanism by which *OAS1* expression in response to infection might, in part, confer resistance to WNV. Five tagSNPs were identified and genotyped in each promoter construct, with clone EcOAS1_A-Luc representing 65.6% and 23.3% of control and case population haplotypes, respectively. Previous studies have shown that expression of the interferon-inducible murine Oas1b during the early stages of WNV infection (6–9 h post-infection) greatly reduced virus production compared to later timepoints [30]. Using transient transfection reporter assays, we investigated potential effects of the promoter mutations on interferon responsiveness. A dose-response was observed for both 2fTGH and HepG2 cells, two cell lines previously utilized in studies of interferon responsiveness and/or *OAS* [25]–[29]. Furthermore, when HepG2 cells were treated with 100 AVU IFN for 6 h, IFN responsiveness was greater in the clone with the common control-population haplotype compared to the others, whose promoter haplotypes were seen more frequently in the case population. Although the differences in fold induction are not dramatic, they are statistically significant and warrant future studies that may help determine how physiological levels of OAS1 expression affect the host response to WNV infection.

WNV infects several tissues during the early viremic phase prior to infection of the central nervous system. As a result, the polymorphisms in the *OAS1* upstream region that are associated with susceptibility to WNV may have a greater functional relevance on *OAS1* expression in other cell lines than those used here. Cell type has been shown to have a pronounced effect on the induction of the p69 isoform of *OAS2* by IFNB [31]. Specifically, expression levels of p69 OAS were substantially higher in lymphoid Daudi cells than in human fibrosarcoma HT1080 cells, the parental cell line of the 2fTGH cells used in these studies [31], [32]. Alternatively, moderate differences in *OAS1* expression may be more readily detectable by quantitative measures of *OAS1* mRNA in WNV-infected cells from horses having either susceptible or resistant genotypes, as was shown in previous studies of WNV-infected mouse embryo fibroblasts [33]. Such infectivity based assays have the advantages of relying on endogenous IFN levels and on the endogenous *OAS1* promoter. The increased sensitivity of such approaches make them more capable of measuring subtle differences in *OAS1* promoter activity that may be important for inhibiting viral replication during the initial stage of infection. Unfortunately, these types of infectivity based assays are not easily implemented in a retrospective case-control study where cases are selected after they have been diagnosed with fatal WNV disease. Therefore, experiments to test the effects of individual *OAS1* promoter variants will have to await the establishment of equine cell lines derived from a variety of tissues that are infected early in WNV disease progression.

While naturally occurring mutations have demonstrated a central role for *Oas* in murine resistance to WNV infection, the radically different composition of the mammalian *OAS* gene clusters make it difficult to extend this conclusion. Our results demonstrate that *OAS1* contributes to naturally occurring WNV susceptibility in a mammal that a) has a very similar *OAS* gene cluster to humans and b) may be more amenable to *in vivo* investigations of the *OAS1* response to WNV infection.

## Methods

### DNA extraction and SNP genotyping of equine samples

Genomic DNA was extracted from white blood cells isolated from whole blood. Control DNA samples were genotyped at each single nucleotide polymorphism as previously described [21]. Case samples consisted of frozen or archived formalin-fixed paraffin-embedded (FFPE) liver, kidney or central nervous (spinal cord or brain) tissues. DNA was extracted from frozen tissue samples after Proteinase K (Promega, Madison, Wisconsin) digestion, washed twice with phenol/chloroform and ethanol precipitated. FFPE liver and kidney samples were deparaffinized with xylene and DNA extracted using the RecoverAll Nucleic Acid Extraction Kit (Ambion, Austin, Texas). FFPE brain and spinal cord samples were deparaffinized with xylene and DNA extracted in a manner similar to frozen samples after treatment with 6 mg Proteinase K for 3 days at 55°C. All FFPE DNA samples were amplified using the Whole Genome Amplification Kit (Sigma, St. Louis, Missouri) using ∼100 ng input DNA without further digestion and amplified for 25 cycles. Amplification products were purified using either the GeneElute Purification System (Sigma, St. Louis, Missouri) or the Qiaquick PCR Purification Kit (Qiagen, Valencia, California). Amplification products from FFPE DNA resulted in fragmented template <500 bp in length (data not shown). FFPE samples were genotyped by sequencing short PCR products <200 bp. PCR primer sequences are available upon request.

### Transfection Experiment

Genotyped samples were amplified with Easy-A high fidelity taq (Stratagene, La Jolla, California) and TA-cloned into pCRII (Invitrogen, Carlsbad, California). Full-length promoters were amplified using PCR primers F:CGACGGCCAGCTCGAGAACCCACAGAATAAACACCACA and R:CAGCTATGACAAGCTTCTGTCAGCCTCTCTCTCTTACG. Primers F:CGACGGCCAGCTCGAGCTTAACCTAGAAACGCGTCTGA and R:CAGCTATGACAAGCTTCTGTCAGCCTCTCTCTCTTACG were used to amplify the 5′ deletion constructs. Individual clones were cultured and verified by sequencing. Each primer pair contains XhoI and HindIII sites used to directionally clone the promoter regions into pGL3-Basic (Promega, Madison, Wisconsin). Final constructs were verified by sequencing (Figure S1).

Human fibrosarcoma 2fTGH cells [32] were maintained in DMEM-F12 medium (Sigma-Aldrich Corp., St. Louis, MO) supplemented with penicillin/streptomycin/amphotericin B (PSA, Invitrogen, Carlsbad, CA) and 5% FBS (Hyclone, Logan,UT). Cells were seeded into 12-well plates, allowed to grow until monolayers were 67–75% confluent and transiently transfected as described previously [34]. Briefly, luciferase constructs (500 ng/well) were co-transfected with an equivalent amount of pEF1-Myc-His LacZ (500 ng/well; Invitrogen) and GenePorter Transfection Reagent (Gene Therapy Systems, San Diego, CA) according to the manufacturer's instructions. Transfected cells were grown overnight (14–16 h) in medium containing 10% FBS before treatment. Recombinant ovine interferon tau (IFNT; 10^8^ antiviral units/mg), a Type I IFN, was produced and assayed as described previously [35]. Transfected cells were treated with 10^2^ to 10^4^ antiviral units (AVU) IFNT/mL or left untreated in serum-free medium. Cells were lysed in Cell Culture Lysis Reagent (Promega, Madison, WI), and luciferase activity (RLU) was assayed according to the manufacturer's instructions (Promega). Human hepatocarcinoma HepG2 cells were grown in DMEM/PSA/10% FBS to 85% confluency before transfection as above except that Lipofectamine 2000 (Invitrogen, Carlsbad, CA) was used at a ratio of 1∶2.5 (DNA∶transfection reagent). HepG2 cells were maintained in complete medium during transfection and subsequent treatment periods.

### Statistical Analysis

Statistical association analyses were conducted using STATA 9 [23] software and the R Statistical Environment [36]. Allelic associations were conducted using Fisher's Exact tests on 2×2 tables. Fisher's Exact tests were conducted on 2×3 tables to identify genotypic associations. Significance is reported with α = 0.05. Haplotype associations were computed using a 2×2 design by comparing single haplotypes to all others. Case-control haplotype frequency analysis was also conducted using Phase v2.

## Supporting Information

Figure S1 Local alignment of human and horse *OAS1* promoters. ClustalX alignment of human (1,036 bp) and equine (1,091 bp) *OAS1* promoters and 5′UTR. Equine *OAS1* was sequenced from CHORI BAC 100:I10 as previously described [21]. Identical sequences are designated with a star (*). The previously identified human interferon-stimulated regulatory element (ISRE) is double-underlined [24]. Significantly associated SNPs are outlined in blue with tagSNPs outlined in red.
(0.02 MB DOC)
