# Supplementary material for: OAS1 Polymorphisms Are Associated with Susceptibility to West Nile Encephalitis in Horses
Source: PLoS One. 2010 May 7;5(5):e10537. doi: 10.1371/journal.pone.0010537 (PMC2866329; doi:10.1371/journal.pone.0010537)
Supplement: Figure S1 — Local alignment of human and horse OAS1 promoters. ClustalX alignment of human (1,036 bp) and equine (1,091 bp) OAS1 promoters and 5′UTR. Equine OAS1 was sequenced from CHORI BAC 100:I10 as previously described [21]. Identical sequences are designated with a star (*). The previously identified human interferon-stimulated regulatory element (ISRE) is double-underlined [24]. Significantly associated SNPs are outlined in blue with tagSNPs outlined in red. (0.02 MB DOC) [file pone.0010537.s001.doc]

Horse AACCCACAGAA-TAAACACCACAA-AGAGAACCCTAATGGGAACTAGAA---------AC

Human AATCCATAGAACTGTAGGACACAAGAGTGAACCTTAATGTAAACCTTAATGTAAATGGAC

** *** **** * * ***** ** ***** ***** *** ** **

EcOAS1_A-Luc AACCCACAGAA-TAAACACCACAA-AGAGAACCCTAATGGGAACCAGAA---------AC

EcOAS1_B-Luc AACCCACAGAA-TAAACACCACAA-AGAGAACCCTAATGGGAACTAGAA---------AC

EcOAS1_C-Luc AACCCACAGAA-TAAACACCACAA-AGAGAACCCTAATGGGAACTAGAA---------AC

Horse TTTAGTTAATAATGATATATCTACAGTCAGGTACCGCTTAACGACGGGGATACGTTCTGA

Human TTTTGTTAATTATGATGTATTAATATCAATTCATCAATTG-TAACAAATGTA---TCACA

*** ****** ***** *** * * * * * ** ** ** ** *

EcOAS1_A-Luc TTTAGTTAATAATGATATATCTACAGTCAGGTACCGCTTAACGATGGGGATACGTTCTGA

EcOAS1_B-Luc TTTAGTTAATAATGATATATCTACAGTCAGGTACCGCTTAACGACGGGGATACGTTCTGA

EcOAS1_C-Luc TTTAGTTAATAATGATATATCTACAGTCAGGTACCGCTTAACGACGGGGATACGTTCTGA

Horse GAAATGCCT-TATTAGGTGATTTTGTCGTTGTTCAAACA---TCATAGCGTTGCTTACAC

Human GTACTGTTAATAATAGAGGAACTTATTGGCAGGAGAGAGAGCTTATGGAACTCTCTGCAC

* * ** ** *** ** ** * * * * ** * * * ***

EcOAS1_A-Luc GAAATGCCT-TATTAGGTGATTTTGTCGTTGTGCAAACA---TCATAGCGTTGCTTACAC

EcOAS1_B-Luc GAAATGCCT-TATTAGGTGATTTTGTCGTTGTTCAAACA---TCATAGCGTTGCTTACAC

EcOAS1_C-Luc GAAATGCCT-TATTAGGTGATTTTGTCGTTGTTCAAACA---TCATAGCGTTGCTTACAC

Horse AAAC--CCGGATGGTAC----AGCCTCCGACACATCTGGACCGTGTGGTACTTATGGGGG

Human ATTCAGCTCAATATTTCTGTAAGCCTAAAACTGCTGTGAGAAATAAAAT-CCAACCTGGG

* * * ** * * ***** ** * ** * * * * ***

EcOAS1_A-Luc AAAC--CCAGATGGCAC----AGCCTCCGACACATCTGGACCGTGTGGTACTTATGGGGG

EcOAS1_B-Luc AAAC--CCGGATGGTAC----AGCCTCCGACACATCTGGACCGTGTGGTACTTATGGGGG

EcOAS1_C-Luc AAAC--CCGGATGGTAC----AGCCTCCGACACATCTGGACCGTGTGGTACTTATGGGGG

Horse CGCCATCGTGT-ATATGGTCTGT-CACTGACTGAAACGTCGTTATTCAGTGCATGACTGT

Human CAACATAGCAAGACCTTGTCTCTACAAAAAATAAAAAATGA--GCTGGGTGCAGTAACGC

* *** * * * **** * ** * * *** * * ***** * *_

EcOAS1_A-Luc CGCCATCGTGT-ATATGGTCTGT-CACTGACTGAAACGTCGTTATTCAGTGCATGACTGT

EcOAS1_B-Luc CGCCATCGTGT-ATATGGTCTGT-CACTGACTGAAACGTCGTTATTCAGTGCATGACTGT

EcOAS1_C-Luc CGCCATCGTGT-ATATGGTCTGT-CACTGACTGAAACGTCGTTATTCAGTGCATGACTGT

Horse ACGTCAATT-CATCAGTTGT--AAAAATATGTACCACGCCAATGTTAATGACAGAAGAAA

Human ATGCCTGTAGTCCCAGGTATTCAGGAGGCTGGGGCAGGAGGATCCCTTGAACCCAGGAAG

* * * * *** * * * * ** ** * ** ** * ***_

EcOAS1_A-Luc ATGTCAATT-CATCAGTTGT--AAAAATATGTACCACGCCAATGTTAATGACAGGAGAAA

EcOAS1_B-Luc ACGTCAATT-CATCAGTTGT--AAAAATATGTACCACGCCAATGTTAATGACAGGAGAAA

EcOAS1_C-Luc ACGTCATTT-CATCAGTTGT--AAAAATATGTACCACGCCAATGTTAATGACAGGAGAAA

Horse TTACGGGTGGAAGGAGAGAGGGCATATG---GGAGTCTGTGCTTTCTGTTCAGTTT---T

Human TTGAGGTTGCACGAGTCATGATCATGCCCCTGCACTCCAGCCTGGATAACAAAGCAAGAT

** ** ** * * * *** * * ** ** * * *

EcOAS1_A-Luc TTACGGGTGGAAGGAGAGGGGGCATATG---GGAGTCTGTGCTTTCTGTTCAGTTT---T

EcOAS1_B-Luc TTACGGGTGGAAGGAGAGGGGGCATATG---GGAGTCTGTGCTTTCTGTTCAGTTT---T

EcOAS1_C-Luc TTACGGGTGGAAGGAGAGGGGGCATATG---GGAGTCTGTGCTTTCTGTTCAGTTT---T

Horse TCTGTAAACATAAAACTGCTGTAAGAAATAATG-TCTAATAATCAAAAGGAAAAAAAGCA

Human CCTGTCTCCAAAAAATAATAAAATAAAATAAAAATCTACTAATTGAAAGGGAAAAAAGCA

**** ** **** * ****** **** **** ***** *********

EcOAS1_A-Luc TCTGTAAACATAAAACTGCTGTAAGAAATAATG-TCTAACAATCAAAAGGAAAAAAAGCA

EcOAS1_B-Luc TCTGTAAACATAAAACTGCTGTAAGAAATAATG-TCTAATAATCAAAAGGAAAAAAAGCA

EcOAS1_C-Luc TCTGTAAACATAAAACTGCTGTAAGAAATAATG-TCTAATAATCAAAAGGAAAAAAAGCA

Horse TAATGGGATGCGATTTTTATAAAACAGAAGAGAGAGCTGTGTGTGTGTGTGTGTGTGTGT

Human TAGTATAATACCATTCTTAACAAAAAGAAAAGAGACCTGTGTTTGTGTGTGTGTTAACAT

** * ** * *** *** *** **** ***** ****** *********** *

EcOAS1_A-Luc TAATGGGATGCCATTTTTATAAAACAGAAGAGAGAGCTGTGTGTGTGTGTGTGTGT----

EcOAS1_B-Luc TAATGGGATGCGATTTTTATAAAACAGAAGAGAGAGCTGTGTGTGTGTGTGTGTGTGTGT

EcOAS1_C-Luc TAATGGGATGCCATTTTTATAAAACAGAAGAGAGAGCTGTGTGTGTGTGTGTGTGTGTGT

Horse GTGTGTGTGT------CTTAACCTAGAAACGCGTCTGAGAAGGCCGGTACCAAGATGTCT

Human TTG-----------------------AAAAAAATCTGGAAAGCTCTATATCAAAACGTTT

** *** **** *** * ** *** * ** *

EcOAS1_A-Luc ----------------CTTAACCTAGAAACGCGTCTGAGAAGGCCGGTACCAAGATGTCT

EcOAS1_B-Luc GTGTGTGTGT------CTTAACCTAGAAACGCGTCTGAGAAGGCCGGTACCAAGATGTCT

EcOAS1_C-Luc GTGTGTGTGTGTGTGTCTTAACCTAGAAACGCGTCTGAGAAGGCCGGTACCAAGATGTCT

Horse GCAGTGGTCGTCTTCGGGTTTGAGGATCGTGGGTGATCTTTACGCTTCCTGATTTTTCTG

Human ATAGAGGCAATTTTGTAGTGTTAGAATCATAGATGATCTTTCCACTTCCTGGTTTTTCTG

** ** * ** ** * ** *** * * ******** * ******* ********

EcOAS1_A-Luc GCAGTGGTCGTCTTCGGGTTTGAGGATCGTGGGTGATCTTTACGCTTCCTGATTTTTCTG

EcOAS1_B-Luc GCAGTGGTCGTCTTCGGGTTTGAGGATCGTGGGTGATCTTTACGCTTCCTGATTTTTCTG

EcOAS1_C-Luc GCAGTGGTCGTCTTCGGGTTTGAGGATCGTGGGTGATCTTTACGCTTCCTGATTTTTCTG

Horse CCTTTTTTCTTTTTCTCA-TATGCACACGCTGCTGTAAAG-ATCATAGCAGACTATAAAA

Human ACTTTTTTTCTTTTTGCAGTGGGCATGTATTGCTGGAAAATACCACAGACAACTGTGAAA

******* **** ** * *** ***** *** * ** ** *** * ***

EcOAS1_A-Luc CCTTTTTTCTTTTTCTCA-TATGCACACGCTGCTGTAAAG-ATCATAGCAGACTATAAAA

EcOAS1_B-Luc CCTTTTTTCTTTTTCTCA-TATGCACACGCTGCTGTAAAG-ATCATAGCAGACTATAAAA

EcOAS1_C-Luc CCTTTTTTCTTTTTCTCA-TATGCACACGCTGCTGTAAAG-ATCATAGCAGACTATAAAA

Horse CAATTTTGTCAGCAACAAAAAAA-GACAAGGAAGGAAATTTAAAAAATCCGTTTTTAATT

Human GGATTTCATCAACAACAAAAAAAAGATAAAGAAGGAAAC--ACAAAATCTG---TTAAAT

**** *** *********** ** ** ******** * ****** * **** *

EcOAS1_A-Luc CAATTTTGCCAGCAACAAAAAAA-GACAAGGAAGGAAATTTAAAAAATCCGTTTTTAATT

EcOAS1_B-Luc CAATTTTGTCAGCAACAAAAAAA-GACAAGGAAGGAAATTTAAAAAATCCGTTTTTAATT

EcOAS1_C-Luc CAATTTTGTCAGCAACAAAAAAA-GACAAGGAAGGAAATTTAAAAAATCCGTTTTTAATT

Horse ATGATTTCTCTTGGATGAGATCCTAATGAGGGTGACAAAGCAACATTTCCCGAGGACAGT

Human AAGATTTATGTTGGCTG-----------GAGGTTAAAATGCA---TTTCCAGAGCAGAGT

* ***** * **** ** *** * ** *** ***** *** * ***

EcOAS1_A-Luc ATGATTTCTCTTGGATGAGATCCTAATGAGGGTGACAAAGCAACATTTTCCGAGGACAGT

EcOAS1_B-Luc ATGATTTCTCTTGGATGAGATCCTAATGAGGGTGACAAAGCAACATTTCCCGAGGACAGT

EcOAS1_C-Luc ATGATTTCTCTTGGATGAGATCCTAATGAGGGTGACAAAGCAACATTTCCCGAGGACAGT

Horse CTGAAGAAAGGCTCGACGCTCCGGGCTGCTGGTTAAAGAACC----GCGAATTTCAGGGA

Human TCAGAGAAAGGCTGG--GCTGCTTGTTGCTGGCTAAAGGACAAAGGGTAAGTTTCAGGAA

********* * *** * * ****** ***** ** * * ******* *

EcOAS1_A-Luc CTGAAGAAAGGCTCGACGCTCCGGGCTGCTGGTTAAAGAACC----GCGAATTTCAGGGA

EcOAS1_B-Luc CTGAAGAAAGGCTCGACGCTCCGGGCTGCTGGTTAAAGAACC----GCGAATTTCAGGGA

EcOAS1_C-Luc CTGAAGAAAGGCTCGACGCTCCGGGCTGCTGGTTAAAGAACC----GCGAATTTCAGGGA

Horse GTGGAGGAACGAGCTGGGAGGGCAGACGCGGCTCAGAGGTGAAAGCAATGTTTGGTTTGC

Human GCAGAAGAGTGAGC----AGATGAAATTCAGCACTGGGATCAGGGGAGTGTCTGATTTGC

* **_** **** ** * * * ** * * * * * * * *** ** *****

EcOAS1_A-Luc GTGGAGGAACGAGCTGGGAGGGCAGACGCGGCTCAGAGGTGAAAGCAATGTTTGGTTTGC

EcOAS1_B-Luc GTGGAGGAACGAGCTGGGAGGGCAGACGCGGCTCAGAGGTGAAAGCAATGTTTGGTTTGC

EcOAS1_C-Luc GTGGAGGAACGAGCTGGGAGGGCAGACGCGGCTCAGAGGTGAAAGCAATGTTTGGTTTGC

Horse TAAGAGGCAAAGGAAACGAAACCAAACGGCAGCCCAGACTTGGAAGACGACTTCCTGCTT

Human -AAAAGGAAAGTGCAA----------------------------AGACAGCTCCTCCCTT

** *** ** * ** **** ** * ***

EcOAS1_A-Luc TAAGAGGCAAAGGAAACGAAACCAAACGGCAGCCCAGACTCGGAAGACGACTTCCTGCTT

EcOAS1_B-Luc TAAGAGGCAAAGGAAACGAAACCAAACGGCAGCCCAGACTTGGAAGACGACTTCCTGCTT

EcOAS1_C-Luc TAAGAGGCAAAGGAAACGAAACCAAACGGCAGCCCAGACTTGGAAGACGACTTCCTGCTT

Horse CCAAGGAAACGAAACCAACAGCAGCCCAGACTCAGGCAACGTAAGAGAGAGAGGCTGACA

Human CTGAGGAAACGAAACCAACAGCAGTCCAAGCTCAGTCAGCAGAAGAGATAAAAGCAAACA

* ********************* *** ***** ** * ****** * * ** ***

EcOAS1_A-Luc CCAAGGAAACGAAACCAACAGCAGCCCAGACTCAGGCAACGTAAGAGAGAGAGGCTGACA

EcOAS1_B-Luc CCAAGGAAACGAAACCAACAGCAGCCCAGACTCAGGCAACGTAAGAGAGAGAGGCTGACA

EcOAS1_C-Luc CCAAGGAAACGAAACCAACAGCAGCCCAGACTCAGGCAACGTAAGAGAGAGAGGCTGACA

Horse GTTTCTGGGAGCCAGTCGTGCAGCCACCAGCTCCTCTGTCCCCAACCGGGCGTCACG

Human GGT-CTGGGAGGCAGTTCTGTTGCCACTCTCTCTCCTGTCA----------------

* * ******* **** ** ***** *** *****_________________

EcOAS1_A-Luc G--------------------------------------------------------

EcOAS1_B-Luc G--------------------------------------------------------

EcOAS1_C-Luc G--------------------------------------------------------

**Supplemental Figure 1. Local Alignment of Human and Horse *OAS1* Promoters**

ClustalX alignment of human (1,036 bp) and equine (1,091 bp) *OAS1* promoters and 5’UTR. Equine OAS1 was sequenced from CHORI BAC 100:I10 as previously described [21]. Identical sequences are designated with star (*). The previously identified human interferon-stimulated regulatory element (ISRE) is double-underlined [24]. Significantly associated SNPs are outlined in blue with tagSNPs outlined in red.
